# Relative fat mass as a novel predictor of gallstone risk: opportunities and caveats

**DOI:** 10.1097/JS9.0000000000002369

**Published:** 2025-04-01

**Authors:** Senda Zhong

**Affiliations:** Anorectal Department, The Public Health Clinical Center of Chengdu, Chengdu, Sichuan Province, China

***Dear Editor***,

We read with great interest the study by Wu *et al*, which evaluates obesity indices and gallstone risk using National Health and Nutrition Examination Survey (NHANES) 2017–2020 data^[^1^]^. Their systematic comparison of traditional (body mass index [BMI], waist circumference) and emerging metrics (Relative Fat Mass [RFM], Body Roundness Index [BRI], Lipid Accumulation Product [LAP]) highlights RFM’s superior discriminative ability (area under the curve [AUC] = 0.70). The proposed cutoff value (RFM ≥ 41.76) effectively identifies high-risk subgroups (women, non-Hispanic Whites, and those with metabolic comorbidities), offering a pragmatic screening tool for primary care. However, we believe three critical issues need to be addressed for successful clinical translation.

## Limited cross-ethnic generalizability

RFM assumes homogeneity in visceral fat distribution across ethnic groups, an assumption that does not reflect biological reality. For example, Asian women exhibit significantly higher visceral adiposity than Caucasian women at equivalent BMIs, with a 29.2% greater visceral fat area after BMI adjustment^[^2^]^. Applying a White-derived threshold (≥41.76) may underdiagnose high-risk Asians and overestimate risk in Africans, who typically have higher subcutaneous fat reserves. We propose a two-phase solution: first, quantify ethnic-specific abdominal fat patterns via imaging (computed tomography [CT]/magnetic resonance imaging [MRI]) to develop ethnic-specific RFM equations, and second, validate these thresholds in independent cohorts to ensure a balance between sensitivity and specificity, ultimately creating dynamic, population-tailored standards.

## Self-report bias and asymptomatic underdiagnosis

The reliance on self-reported gallstone diagnoses introduces two potential biases. Recall bias may lead patients to misattribute symptoms (e.g., confusing gallstones with gastroesophageal reflux disease [GERD]) or forget asymptomatic cases, which have a prevalence of approximately 20%. This could skew false-positive/negative rates. Asymptomatic individuals, who are often undiagnosed due to the absence of medical encounters, are systematically excluded from the study, which may underestimate the true predictive power of RFM. Moreover, obesity accelerates the transition from asymptomatic to symptomatic disease, which could distort RFM’s risk stratification, particularly in high-burden populations. To improve diagnostic rigor, we recommend that future studies incorporate baseline ultrasound screening (defining gallstones by echogenic foci with acoustic shadows) in prospective cohorts, along with longitudinal follow-up of asymptomatic individuals to capture dynamic risk progression.

## Unaccounted gene-obesity interactions

Although metabolic confounders were adjusted for, the study did not consider the role of genetic predisposition and its interactions with obesity. For instance, the ATP-binding cassette subfamily G member 8 (ABCG8) rs11887534 variant independently increases gallstone risk^[^3^]^ by impairing biliary cholesterol excretion – an effect unrelated to BMI. Carriers of this variant may develop gallstones despite a low RFM, while non-carriers with high RFM might experience lower-than-expected risks. Furthermore, gene-environment interactions could amplify risk: RFM-driven insulin resistance upregulates 3-hydroxy-3-methylglutaryl-coenzyme A (HMG-CoA) reductase activity, accelerating cholesterol synthesis – a pathway that is potentiated in genetically susceptible individuals. Ignoring genetic heterogeneity introduces unmeasured confounding, which could compromise precision prevention. We recommend incorporating key genetic variants into multivariate models to quantify the impact of RFM across different genotypes, enabling more tailored prevention strategies.

In conclusion, we recommend: (1) the development of dynamically calibrated RFM equations using multiethnic imaging data; (2) validation of screening efficacy in ultrasound-confirmed prospective cohorts; and (3) the construction of refined models integrating genetic biomarkers. Dr. Wu’s study represents a significant step toward improving gallstone prevention in obesity-endemic settings. Addressing these challenges will help bridge the gap between research and clinical implementation.

## Data Availability

All data used in this correspondence are publicly available from the U.S. CDC NHANES database (2017–2020 cycle) at: https://www.cdc.gov/nchs/nhanes/.
